# Frontiers of Targeted Therapy and Predictors of Treatment Response in Systemic Sclerosis

**DOI:** 10.3390/biomedicines10123053

**Published:** 2022-11-28

**Authors:** Michal Tomcik

**Affiliations:** Institute of Rheumatology, Department of Rheumatology of the First Faculty of Medicine, Charles University, Na Slupi 4, 128 00 Prague, Czech Republic; tomcik@revma.cz

Systemic sclerosis (scleroderma, SSc) is one of the most challenging rheumatic diseases, characterized by vasculopathy, dysregulation of the immune response, and progressive tissue fibrosis affecting the skin, lungs, heart, digestive tract, and kidneys. To date, it is considered incurable and carries the highest cause-specific mortality of all connective tissue diseases. This is especially true in the later stages of the disease, when irreversible damage to the musculoskeletal system and internal organs prevails, which significantly reduces overall function, the ability to perform activities of daily living, and the quality of life. Despite advances in basic, translational, and clinical research in recent years, the etiology and pathophysiology of this complex condition remain to be elucidated. In spite of a large number of clinical trials and the progress made in their design over the last decade, no approved disease-modifying therapies exist for SSc to date. Currently available pharmacological therapies predominantly target inflammatory and vascular pathways, have variable and unpredictable clinical efficacy and usually undesirable safety profiles, and only have a modest effect on long-term survival [1]. 

Therefore, the focus of several research groups has been to unravel novel therapeutic targets driving the main pathological processes of this disease and to identify potential predictors of the treatment response of the currently available therapies, in order to optimize the stratification of patients with this heterogeneous disease while minimizing the possible adverse events and maximizing the potential benefit of therapy. The collection of six original research manuscripts and five review articles highlights the most recent progress in this field and provides further elucidation of the pathogenesis and natural disease course of this enigmatic disease. 

A large body of evidence supports the concept that the activation of the immune system drives the complex interplay between vasculopathy and fibrogenesis in SSc. In their review article, Papadimitriou et al. focus on the contribution of the innate and acquired immune system to the development and progression of tissue fibrosis in SSc [2]. They provide a complex overview of crucial cellular and soluble immune mediators implicated in SSc pathogenesis and discuss their impact on skin and lung fibrosis. Furthermore, they review the evidence supporting the use of first-line anti-inflammatory therapies (e.g., corticosteroids, methotrexate, cyclophosphamide, mycophenolate mofetil, and autologous hematopoietic stem cell transplantation), T cell-, B cell-, and cytokine-specific targeted therapies (e.g., rituximab, inebilizumab, belimumab, abatacept, tocilizumab, rilonacept, basiliximab, fresolimumab, metelimumab, and pirfenidone), as well as emerging therapies with tyrosine kinase inhibitors (e.g., imatinib mesylate, nilotinib, nintedanib, and tofacitinib) to reduce fibrosis.

Among the plethora of soluble immune mediators implicated in the pathogenesis of SSc, interleukin (IL)-6 plays a vital role both in microangiopathy and the development of tissue fibrosis. Cardoneanu et al. provide an overview of the involvement of IL-6 in the activation and apoptosis of vascular endothelial cells, the potentiation and maintenance of innate and adaptive immune response, and fibrogenesis [3]. They discuss the systemic adverse effects of IL-6 in the involvement of the skin, lungs, kidneys, gastrointestinal tract, and cardiovascular system, and present a comprehensive overview of the published data on the clinical efficacy of tocilizumab in the treatment of SSc-related manifestations.

All three of the above-mentioned processes involved in the pathogenesis of SSc can also be modulated by epigenetic mechanisms. In their review article, Szabo et al. provide an extensive overview of complex interactions of micro RNAs (miRNAs, small non-coding RNA sequences) with microangiopathy, immune activation, and tissue fibrosis [4]. They present the published serum- and tissue-specific miRNA signatures in SSc and focus on miRNA transcripts with pro- and anti-fibrotic properties, and the roles of miRNAs in fibroblast apoptosis and the pathogenesis of SSc-related interstitial lung disease (ILD), vascular damage and immune dysfunction. Furthermore, they discuss the potential use of miRNAs as diagnostic and prognostic biomarkers as well as potential novel targeted therapies of SSc.

Zaaroor Levy et al. studied the circulating miRNAs that may differentiate between SSc patients with and without pulmonary arterial hypertension (PAH) and assessed their expression in plasma, white blood cells, and dermal myofibroblasts [5]. They demonstrate that plasma levels of miR-26 and miR-let-7d are decreased in SSc-PAH patients compared to SSc patients without PAH. Furthermore, their findings suggest that miR-26 and miR-let-7d might be involved in the activation of fibroblasts by regulating the genes in the transforming growth factor (TGF)-β and endothelin-1 pathways, and their reduced plasma levels could be an early predictor of the development of PAH.

In their original article, Štorkánová et al. present compelling evidence for a potential novel therapeutic target implicated in SSc-related skin fibrosis [6]. Using a modified murine model of bleomycin-induced dermal fibrosis, they demonstrate that the inhibition of Heat shock protein (Hsp)-90 by 17-dimethylaminoethylamino-17-demethoxy-geldanamycin (17-DMAG) prevents further progression and may induce regression of the established skin fibrosis with a comparable effect to that of nintedanib. The anti-fibrotic effects of 17-DMAG were mediated by the inhibition of fibroblast activation, suppression of TGF-β/Smad signaling, and the systemic and local inflammatory response induced by bleomycin. Since several Hsp90 inhibitors have been assessed in clinical trials for oncological indications, these preclinical findings may have translational implications in the treatment of SSc-related tissue fibrosis.

Bögl et al. used targeted and untargeted metabolomic approaches to elucidate the distinct biochemical mechanisms that might be relevant to the pathophysiology of SSc [7]. Their metabolomic profiling in plasma from SSc patients compared to healthy controls revealed four significantly dysregulated metabolic pathways, namely the kynurenine pathway, urea cycle, lipid metabolism, and gut microbiome, all of which are associated with autoimmune inflammation, vascular damage, fibrosis, and intestinal dysbiosis. These four altered metabolomics networks deserve further validation; however, they could become potential therapeutic targets or biomarkers of treatment response in SSc.

The potential pathomechanisms leading to the maturation of autoreactive B cells, formation of autoantibodies, and processes underlying the potential promotion and maintenance of pathologic processes involved in SSc are not sufficiently understood. These issues are extensively discussed in the review article of Graßhoff and others [8]. They also provide a comprehensive overview of autoantibodies which might be useful as biological predictors of disease course and of therapeutic approaches that have been or might be used in the future for the treatment of autoantibody-induced pathologies in SSc. 

Recent advances in the basic and clinical research on ILD, as a leading cause of mortality in SSc, have led to the discovery of novel molecular therapeutic targets. The review article by Aragona et al. analyzes phase II/III randomized clinical trials focused on SSc-ILD, which were published between January 2016 and December 2021 [9]. The authors provide supporting evidence on the phase III trials involving nintedanib, tocilizumab, and lenabasum, as well as of phase II trials involving pirfenidone, pomalidomide, romilkimab, riociguat, rituximab, and abatacept. In addition, they report on the head-to-head studies of rituximab vs. cyclophosphamide and mycophenolate mofetil vs. cyclophosphamide, as well as future directions in clinical trials on SSc-ILD. 

Two studies from the research group of Veronika Müller shed some light on the disease course of SSc-ILD from two different perspectives. Nagy T et al. examined SSc-ILD patients with a normal initial spirometry and followed up with them for at least one year [10]. They reported that patients who present with cough and pulmonary hypertension were at a higher risk of developing progressive decline in lung functions. Similarly, Nagy A et al. assessed the disease course and progressive functional decline in SSc-ILD patients treated in a real-world setting and demonstrated that the most prominent functional decline occurred in untreated patients, which was as expected, but paradoxically, in those with BMI < 25 kg/m^2^ [11]. Thus, overweight SSc-ILD patients appear to be at a lower risk of progressive functional decline. These predictors require further validation; however, they might be useful for defining a high-risk population of patients, who should be closely monitored with regular follow-ups and started early and aggressively on immunosuppressive and/or antifibrotic therapy.

In the last original article, Rauch et al. address the unmet need for new treatment options for a therapeutically problematic clinical manifestation of SSc, cutaneous calcinosis [12]. They retrospectively assessed the effect of a second-generation bisphosphonate, intravenous pamidronate, on cutaneous calcinosis, and demonstrated a reduction in pain, improvement in general condition, and cessation of progression in 83%, regression of calcinosis in 33% of patients, stable disease in 17% of patients, and radiological improvement or stabilization in 50% of the patients. Fever was the most common side effect and one patient developed jaw osteonecrosis.

Hopefully, the novel advancements included here covering several aspects of the pathogenesis, prognosis, and management of SSc will contribute to a reduction in suffering and disability, as well as an improvement in the morbidity and mortality of our patients in the near future.
